# Genome Wide Association Studies for Milk Production Traits in Chinese Holstein Population

**DOI:** 10.1371/journal.pone.0013661

**Published:** 2010-10-27

**Authors:** Li Jiang, Jianfeng Liu, Dongxiao Sun, Peipei Ma, Xiangdong Ding, Ying Yu, Qin Zhang

**Affiliations:** Key Laboratory of Animal Genetics and Breeding of the Ministry of Agriculture, College of Animal Science and Technology, China Agricultural University, Beijing, People's Republic of China; Aarhus University, Denmark

## Abstract

Genome-wide association studies (GWAS) based on high throughput SNP genotyping technologies open a broad avenue for exploring genes associated with milk production traits in dairy cattle. Motivated by pinpointing novel quantitative trait nucleotide (QTN) across *Bos Taurus* genome, the present study is to perform GWAS to identify genes affecting milk production traits using current state-of-the-art SNP genotyping technology, i.e., the Illumina BovineSNP50 BeadChip. In the analyses, the five most commonly evaluated milk production traits are involved, including milk yield (MY), milk fat yield (FY), milk protein yield (PY), milk fat percentage (FP) and milk protein percentage (PP). Estimated breeding values (EBVs) of 2,093 daughters from 14 paternal half-sib families are considered as phenotypes within the framework of a daughter design. Association tests between each trait and the 54K SNPs are achieved via two different analysis approaches, a paternal transmission disequilibrium test (TDT)-based approach (L1-TDT) and a mixed model based regression analysis (MMRA). In total, 105 SNPs were detected to be significantly associated genome-wise with one or multiple milk production traits. Of the 105 SNPs, 38 were commonly detected by both methods, while four and 63 were solely detected by L1-TDT and MMRA, respectively. The majority (86 out of 105) of the significant SNPs is located within the reported QTL regions and some are within or close to the reported candidate genes. In particular, two SNPs, ARS-BFGL-NGS-4939 and BFGL-NGS-118998, are located close to the *DGAT1* gene (160bp apart) and within the *GHR* gene, respectively. Our findings herein not only provide confirmatory evidences for previously findings, but also explore a suite of novel SNPs associated with milk production traits, and thus form a solid basis for eventually unraveling the causal mutations for milk production traits in dairy cattle.

## Introduction

Over the last decades, advances in DNA-based marker technology make it possible to identify genome regions (namely quantitative trait loci, QTL) underlying complex traits such as milk yield in dairy cattle. Instead of traditional animal breeding programmes solely relying on phenotype and pedigree information, the incorporation of detected QTL into genetic evaluation provides a great potential to enhance selection accuracies, hence expediting the genetic improvement of animal productivity.

In dairy cattle, since the seminal work on QTL mapping by Georges el al [1], a large number of articles have been published concerning detection of QTLs for milk production traits. So far a total number of 1,137 QTL for milk production traits have been reported via genome scan based on marker-QTL linkage analyses (http://www.animalgenome.org/QTLdb/cattle.html, May 22, 2010). The limitations of QTL mapping using linkage analysis (LA) and/or linkage disequilibrium (LD) [2] based on panels of low to moderate density markers have been well documented previously [3], [4]. In the past decades merely few strong candidate genes with potential effects on milk production traits, *i.e.*, the *DGAT1* gene [5] and the *GHR* gene [6], have been identified and/or functionally confirmed from those findings derived from QTL linkage analyses and fine mapping studies.

With the advent of genome-wide panels of single nucleotide polymorphisms (SNPs), SNPs have been widely used for the detection and localization of QTL for complex traits in many species [7], and have proved powerful and useful in identification of casual mutations associated with economically important traits in livestock [8], [9], [10], [11], [12], [13], [14], [15] as well as human diseases [16], [17], [18], [19]. Most recently, along with maturing of genome sequencing and high throughput SNP genotyping technologies, genome-wide association studies (GWAS) are becoming practical for exploring genes associated with complex traits. Compared with traditional QTL mapping strategy, GWAS brings on major advantages both in power to detect causal variants with modest effects and in defining narrower genomic regions harboring causal variants [20]. GWAS has been widely accepted as a primary approach for gene finding and achieved huge success in identifying genes conferring modest disease risks in human. However, only few GWAS focusing on identifying genes for milk production traits have been performed [21], [22]. Furthermore, the common limitation of these studies is that low-density SNP makers were employed in the analyses, leading to a decrease in power to capturing causal genes.

Motivated by searching for novel casual variants for milk production traits beyond previous findings via traditional linkage studies, the present study is to perform GWAS to detect potential casual genetic variants for milk production traits, using the Illumina BovineSNP50 BeadChip. The identified SNP loci may be considered as preliminary foundation for further replication studies and eventually unraveling the causal mutations for milk production traits in dairy cattle.

## Materials and Methods

The blood samples were collected along with the regular quarantine inspection of the farms, so no ethical approval was required for this study.

### Animal resource

A daughter design was employed in this study. In total 2,093 daughters as well as their 14 corresponding sires were collected to construct the study population. The numbers of daughters of the 14 sires range from 83 to 358 daughters with an average of 150. These daughters were from 15 Holstein cattle farms in Beijing, China, where regular and standard performance testing (dairy herd improvement, DHI) has been conducted since 1999. The official up to date estimated breeding values (EBVs) of five milk production traits, including milk yield (MY), fat yield (FY), protein yield (PY), fat percentage (FP), and protein percentage (PP) were used as phenotypes in this study. These EBVs were obtained based on a multiple trait random regression test-day model [23] using the software RUNGE provided by Canadian Dairy Network (CDN) (http://www.cdn.ca). The descriptive statistics of these EBVs for the five traits as well as the average reliabilities of EBVs of the 2,093 daughters are presented in Table 1. It is notable that the program RUNGE gave two sets of accuracies of EBVs for the five milk production traits. One is for milk yield (MY) and the other for the four milk content traits (FY, PY, FP, and PP). This is because that the amount of information used for calculating EBVs was different for MY and for the 4 milk content traits, while all of the 4 milk content traits provided the same amount of information for calculating EBVs.

**Table 1:** Descriptive statistics of EBVs and the accuracy of five milk production traits for 2,093 daughters.

Traits	Mean	Standard deviation	Minimum	Maximum	Mean reliability (range)
Milk yield (MY)	379.36	608.65	−1667.00	2553.00	0.63 (0.50–0.71)
Fat yield (FY)	7.49	24.37	−73	94	0.52 (0.41–0.70)
Protein yield (PY)	10.72	17.05	−49	74	0.52 (0.41–0.70)
Fat% (FP)	−0.07	0.91	−0.90	0.27	0.52 (0.41–0.70)
Protein% (PP)	−0.01	0.32	−0.42	0.10	0.52 (0.41–0.70)

### Genotyping

DNA was extracted from blood sample of the daughters and semen sample of the sires using the routine procedures. DNA was quantified and genotyped using the Illumina BovineSNP50 BeadChip containing 54001 SNPs, which is a multi-sample genotyping panel powered by Illumina's Infinium® II Assay. Features of the Illumina BovineSNP50 BeadChip have been detailed previously [24]. All samples were genotyped using BEADSTUDIO (Illumina) and a custom cluster file developed from the 2180 samples.

### Genotype quality control

To assess the technical reliability of the genotyping panel, a randomly selected DNA sample was genotyped twice and over 99% identity of called genotypes (two mismatches) was obtained. This demonstrates the technically robust feature of the 50K SNP BeadChip panel employed herein.

The quality control procedure can be largely split into two categories, including individual exclusion and SNP removal, as follows:

Firstly, an individual would be excluded from the analyses if it had more than 10% missing genotypes or its SNP genotypes had a Mendelian error rate above 2%. For the second criterion, for each sire-daughter pair, we randomly choose 10,000 genotyped SNP loci for which both the sire and the daughter are homozygotes. A Mendelian error happens herein if the two homozygotes are different in the context that the maternal genotype is unavailable. Accordingly, if more than 200 out of 10,000 SNP have Mendelian errors, the daughter will be removed from the sample.

Secondly, a SNP would be removed if (1) its call rate was less than 90%, or (2) its minor allele frequency (MAF) was less than 3%, or (3) it was severely depart from Hardy Weinberg Equilibrium (HWE) with a *P* value lower than 10^−6^, or (4) its minor genotype frequency was less than five individuals.

After the quality control procedures, 73 daughters with >10% missing genotypes and 205 daughters with Mendelian error rate above 2% were excluded, leading to 1,815 daughters remaining for the association analysis. On the other hand, we removed 1,218 SNPs with <90% genotype call rate, 11,008 SNPs with a MAF <0.03, 482 SNPs with extreme value of HWE statistics (*P*<10^−06^), and 1,073 SNPs with minor genotype frequency <5 individuals. Eventually, 40,220 SNPs (74.5%) passed these quality control filters. The distribution of the remaining SNPs after filtering and the average distances between adjacent SNPs on each chromosome are given in Table 2. In addition, for the L1-TDT analyses, we excluded extra 1,057 SNPs for which all paternal genotypes are homozygotes, and 39,163 SNPs were finally utilized.

**Table 2:** Distributions of SNPs after quality control and the average distances between adjacent SNPs on each chromosome.

BTA	No. SNPs	Average distance (kb)^a^
1	2485	65
2	2033	69
3	1966	65
4	1869	66
5	1607	78
6	1913	64
7	1711	65
8	1797	65
9	1499	72
10	1586	67
11	1699	65
12	1233	69
13	1318	64
14	1309	62
15	1265	67
16	1180	66
17	1201	64
18	1039	64
19	1068	61
20	1205	63
21	1053	66
22	939	66
23	838	63
24	946	69
25	769	57
26	774	67
27	730	67
28	719	64
29	794	65
X	497	179
0^b^	1178	
TOTAL	40220	

a Derived from the most recent bovine genome sequence assembly (Btau_4.0) (http://www.ncbi.nlm.nih.gov/genome/guide/cow/)

b These SNPs are not assigned to any chromosomes.

### Statistical analyses

Two methods are adopted to perform GWAS in our studies as follows:

#### TDT-based single locus regression analyses (L1-TDT)

L1-TDT is a TDT-based association procedure [25], which is specifically suitable for the situation where only a single parent instead of both parents are genotyped for TDT analyses. As merely the genotypes of bulls and their daughters are available within the framework of a daughter design, we employed it to explore the existence of associations between phenotypes and SNP allele transmissions from bulls to their daughters within sire families. Under such circumstance, a phenotypic observation, *i.e.*, EBV considered herein, can be modeled by a SNP effect within family due to transmission disequilibrium of the SNP alleles as well as the effect of the sire corresponding to each half-sib family. For each milk production trait, the equation of the model is given as follows:
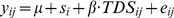
(1)where *y_ij_* is the EBV of the *j* ^th^ daughter of sire *i*, *μ* is the overall mean, *s_i_* is the fixed effect of sire *i*, *TDS_ij_* is an indicator variable with a value −1, 0 or 1 to indicate the transmission of a specific SNP allele from sire *i* to his *j* ^th^ daughter, which is determined according to [26], *β* is the regression coefficient (or the substitution effect of the SNP), and *e_ij_* is the residual error. For each SNP, *β* is estimated via a weighted least squares analysis with the weights equal to 1/*REL_ij_*, where *REL_ij_* is the reliability of the EBV of daughter *j* in family *i*. The association between the SNP and the trait is tested via the *F*-test.

#### Mixed model based single locus regression analyses (MMRA)

Similar to the studies of [21] and [22], we performed association test for each SNP via regression analysis based on the following linear mixed model:
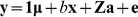
(2)where **y** is the vector of EBVs of all daughters, *b* is the regression coefficient of EBV on SNP genotypes, **x** is the vector of the SNP genotype indicators which takes values 0, 1 or 2 corresponding to the three genotypes 11, 12 and 22 (assuming 2 is the allele with a minor frequency), **a** is the vector of the residual polygenetic effects with 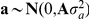 (where **A** is the additive genetic relationship matrix and 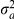 is the additive variance, and e is the vector of residual errors with 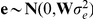 (where **W** is a diagonal matrix with the diagonal elements equal to 1/REL*_ij_* and 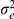 is the residual error variance). For each SNP, the estimate of *b* and the corresponding sampling variance 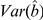 can be obtained via mixed model equations (MME), and a Wald chi-squared statistic 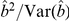 with *df* = 1 is constructed to examine whether the SNP is associated with the trait.

We employed Fortran 95 to code the computing programs for L1-TDT and MMRA and they are available upon request.

### Statistical Inference

For both analyses, the Bonferroni method was adopted to adjust for multiple testing from the number of SNP loci detected. We declared a significant SNP at the genome-wise significance level if a raw *P* value <0.05/*N*, here *N* is the number of SNP loci tested in analyses.

### Population stratification assessment

Confounding due to population stratification has been considered as a major plague to the validity of genetic association studies [27]. To view if the population stratification exists in our experimental population, we examined the distribution of the test statistics obtained from the numerous association tests performed and assessed their deviation from the expected distribution of no SNP being associated with the trait of interest utilizing a quantile-quantile (Q-Q) plot, which is a routine and most frequently used tool for scrutinizing the population stratification in GWAS. Since merely MMRA method is not immune to potential population stratification, “Q-Q” plots for the test statistics of MMRA were conducted for the five traits.

## Results

### Significant SNPs

**Figure 1:**
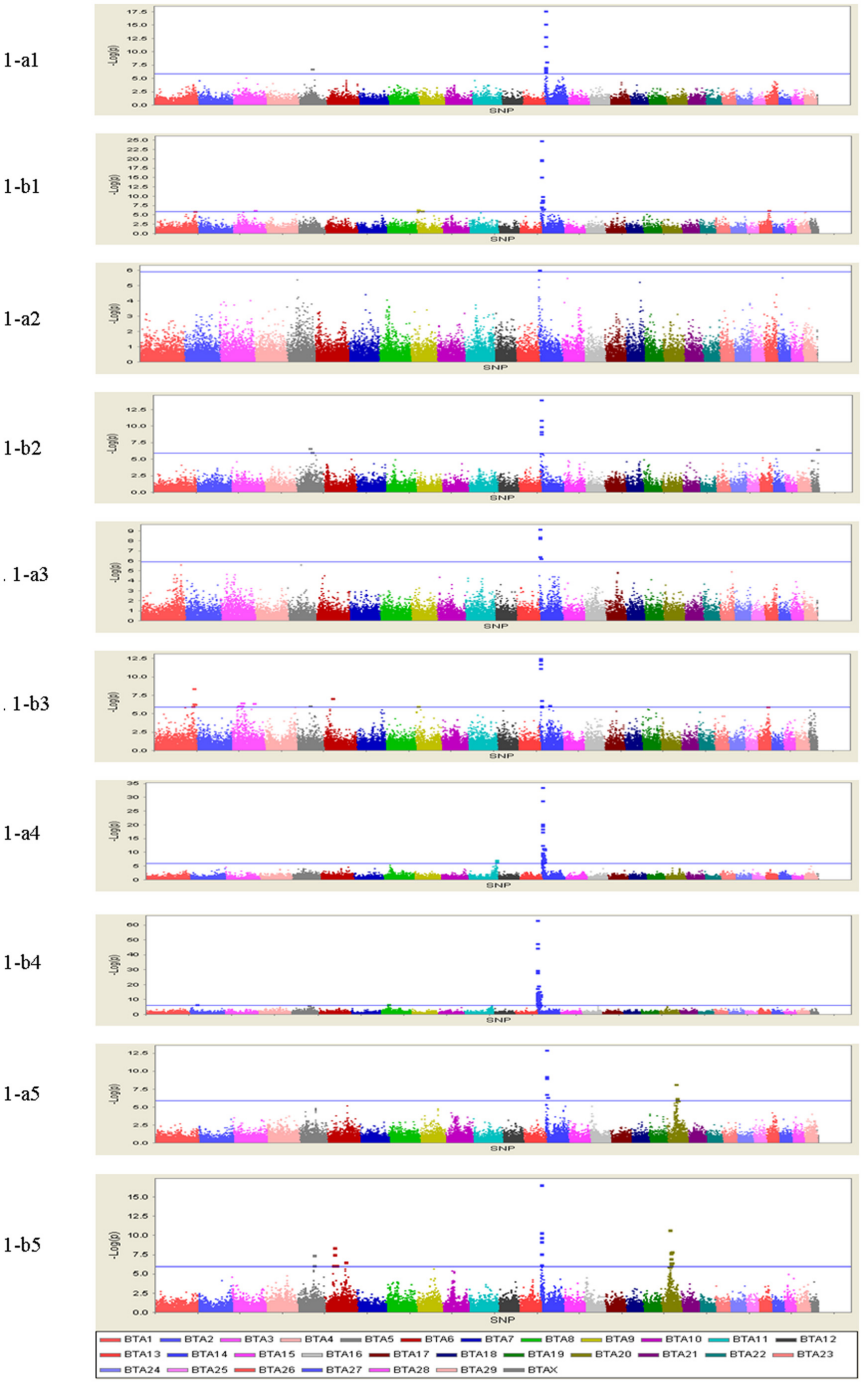
Genome-wide plots of −log_10_(*p*-values) for association of SNP loci with five milk production traits in sequential order. Chromosomes 1–29 and X are shown separated by color. Fig. 1-a1, 1-a2, 1-a3, 1-a4 and 1-a5 refer to plots generated by L1-TDT for MY, FY, PY, FP and PP, respectively. Fig. 1-b1, 1-b2, 1-b3, 1-b4 and 1-b5 refer to plots generated by MMRA for MY, FY, PY, FP and PP, respectively. The corresponding horizontal lines indicate the genome-wise significance levels (−log_10_(1.28×10^−6^) for L1-TDT and −log_10_(1.24×10^−6^) for MMRA).

The profiles of *P* values (in terms of −log(*p*)) of all tested SNPs for the five investigated traits are shown in Fig. 1. The numbers of genome-wise significant SNPs detected by L1-TDT or MMRA for the five traits are presented in Table 3. In total, the numbers of significant SNPs detected by either L1-TDT or MMRA for the five traits are 20, 9, 21, 65 and 28, respectively. Since some of these SNPs are associated with more than one trait, the total number of distinct identified SNPs is 105. Of these 105 SNPs, 38 were commonly detected by both methods, while four and 63 were solely detected by L1-TDT and MMRA, respectively. With exception of only four SNPs, all SNPs detected by L1-TDT were also detected by MMRA. The details of these significant SNPs for the five traits, including their positions in the genome, the nearest known genes and the raw *P* values, are given in Tables 4 through [Table pone-0013661-t005] [Table pone-0013661-t006] [Table pone-0013661-t007] 8, respectively, and further described as follows.

**Table 3:** Numbers of significant SNPs detected by L1-TDT and MMRA.

Trait	L1-TDT	MMRA	Overlap^a^	Total^b^
MY	11	18	9	20
FY	1	9	1	9
PY	5	21	5	21
FP	37	61	33	65
PP	10	27	9	28

a Numbers of SNPs commonly detected by both methods.

b Numbers of SNPs detected by either L1-TDT or MMRA.

**Table 4:** Genome-wise significant (*p*<0.05) SNPs for milk yield (MY).

SNP	Chr.	Position (bp)	Nearest gene^c^		Raw *P* value	Adjusted *P* value
			Name	Distance (bp)		
ARS-BFGL-NGS-91705^b^ (rs43282015)	1	149650628	*LOC614166*	5490	9.21E-07	3.70E-02
Hapmap38643-BTA-95454^b^	3	92862402	*LOC534011*	Within	6.24E-07	2.51E-02
BFGL-NGS-110018^a^ (rs41647754)	5	64833594	*HAL*	357	1.50E-07	5.89E-03
ARS-BFGL-NGS-49079^b^ (rs42517915)	9	5763632	*LOC788012*	56757	3.53E-07	1.42E-02
*BTB-01921442* ^b^ (rs43030751)	9	21062122	*LOC100139865*	30952	6.61E-07	2.66E-02
Hapmap30383-BTC-005848^ab^	14	76703	*C14H8orf33*	87	7.58E-16	3.05E-11
*ARS-BFGL-NGS-57820* ^ab^	14	236532	*FOXH1*	3396	1.39E-20	5.59E-16
*ARS-BFGL-NGS-34135* ^ab^	14	260341	*CYHR1*	Within	6.18E-08	2.49E-03
*ARS-BFGL-NGS-94706* ^b^ (rs17870736)	14	281533	*VPS28*	Within	4.08E-09	1.64E-04
*ARS-BFGL-NGS-4939* ^ab^	14	443937	*DGAT1*	160	1.16E-25	4.67E-21
*Hapmap52798-ss46526455* ^a^ (rs41256919)	14	565311	*MAF1*	Within	8.22E-08	3.22E-03
*ARS-BFGL-NGS-107379* ^ab^	14	679600	*LOC786966*	460	2.33E-20	9.37E-16
UA-IFASA-6878^ab^ (rs41629750)	14	1044041	*GRINA*	15662	8.74E-08	3.52E-03
Hapmap25486-BTC-072553^b^	14	1285037	*GML*	Within	3.32E-07	1.34E-02
Hapmap30646-BTC-002054^ab^	14	1461085	*GPIHBP1*	1295	1.02E-10	4.10E-06
Hapmap30086-BTC-002066^ab^	14	1490178	*ZNF66*	1566	7.74E-10	3.11E-05
*ARS-BFGL-NGS-100480* ^ab^	14	2607583	*NIBP*	Within	2.91E-09	1.17E-04
*UA-IFASA-6329* ^b^ (rs41579243)	14	3465237	*COL22A1*	9864	1.59E-09	6.39E-05
*BFGL-NGS-110563* ^b^	14	3799228	*COL22A1*	84554	2.25E-07	9.05E-03
*Hapmap50053-BTA-61516* ^b^	26	39018261	*C26H10orf84*	28724	4.83E-07	1.94E-02

Note: SNPs with a superscript “a” are detected by L1-TDT only, SNPS with a superscript “b” are detected by MMRA only, SNPs with a superscript “ab” are detected by both L1-TDT and MMRA, and SNPs in italic are located within the QTL regions reported previously. Names in parentheses are standard RRS/RS names in the NCBI database (http://www.ncbi.nlm.nih.gov).

c The nearest known gene to the significant SNP.

**Table 5 pone-0013661-t005:** Genome-wise significant (*p*<0.05) SNPs with fat yield (FY).

SNP	Chr.	Position (bp)	Nearest gene		Raw *P* value	Adjusted *P* value
			Name	Distance (bp)		
*Hapmap57440-rs29017368* ^b^ (rs29017368)	5	62242550	*LOC515967*	2419	2.22E-07	8.93E-03
*Hapmap40191-BTA-73919* ^b^ (rs41648982)	5	76882812	*LOC511240*	69845	8.10E-07	3.26E-02
*Hapmap30381-BTC-005750* ^b^	14	50872	*C14H8orf33*	23701	1.14E-06	4.59E-02
*ARS-BFGL-NGS-57820* ^b^	14	236532	*FOXH1*	3396	1.23E-10	4.95E-06
*ARS-BFGL-NGS-34135* ^b^	14	260341	*CYHR1*	Within	1.29E-11	5.19E-07
*ARS-BFGL-NGS-94706* ^ab^ (rs17870736)	14	281533	*VPS28*	Within	8.93E-07	3.50E-02
*ARS-BFGL-NGS-4939* ^b^	14	443937	*DGAT1*	160	1.01E-14	4.06E-10
*ARS-BFGL-NGS-107379* ^b^	14	679600	*LOC786966*	460	1.47E-09	5.91E-05
BTA-90435-no-rs^b^ (rs41664719)	X	70532837	*LOC516454*	285289	3.35E-07	1.35E-02

Note: See note to Table 4.

**Table 6 pone-0013661-t006:** Genome-wise significant (p<0.05) SNPs with protein yield (PY).

SNP	Chr.	Position (bp)	Nearest gene		Raw *P* value	Adjusted *P* value
			Name	Distance (bp)		
*BTA-55340-no-rs* ^b^ (rs41586699)	1	145954149	*PDE9A*	Within	9.72E-07	3.91E-02
BFGL-NGS-113002^b^	1	149153073	*DIP2A*	Within	4.56E-07	1.83E-02
ARS-BFGL-NGS-91705^b^ (rs43282015)	1	149650628	*LOC614166*	5490	3.57E-09	1.44E-04
*ARS-BFGL-NGS-98387* ^b^	1	154783580	*ETS2*	169745	4.93E-07	1.98E-02
*INRA-701* ^b^ (*rs41589462*)	3	33837442	*LOC539739*	Within	7.75E-07	3.12E-02
*BFGL-NGS-115461* ^b^	3	45261895	*SLC30A7*	7306	8.47E-07	3.41E-02
*Hapmap58769-rs29025951* ^b^ (rs29025951)	3	47803786	*LOC100138725*	219493	3.11E-07	1.25E-02
ARS-BFGL-NGS-4358^b^	3	50781421	*LOC781902*	385764	3.04E-07	1.22E-02
Hapmap38643-BTA-95454^b^	3	92862402	*LOC534011*	Within	3.77E-07	1.52E-02
*BFGL-NGS-110018* ^b^ (rs41647754)	5	64833594	*HAL*	357	8.53E-07	3.43E-02
*ARS-BFGL-NGS-7249* ^b^	6	30503076	*PDHA2*	13265	8.38E-08	3.37E-03
*BTA-83825-no-rs* ^b^ (rs41659807)	9	7704446	*LOC788115*	60854	9.79E-07	3.94E-02
Hapmap30383-BTC-005848^ab^	14	76703	*C14H8orf33*	87	2.99E-13	1.20E-08
*ARS-BFGL-NGS-57820* ^ab^	14	236532	*FOXH1*	3396	1.71E-12	6.88E-08
*ARS-BFGL-NGS-4939* ^ab^	14	443937	*DGAT1*	160	5.81E-13	2.34E-08
*ARS-BFGL-NGS-107379* ^ab^	14	679600	*LOC786966*	460	6.18E-12	2.49E-07
*Hapmap30646-BTC-002054* ^b^	14	1461085	*GPIHBP1*	1295	1.55E-07	6.23E-03
*ARS-BFGL-NGS-100480* ^ab^	14	2607583	*NIBP*	Within	1.16E-06	4.67E-02
*UA-IFASA-6329* ^b^ (rs41579243)	14	3465237	*COL22A1*	9864	7.59E-07	3.05E-02
*ARS-BFGL-BAC-10793* ^b^	14	27452257	*NKAIN3*	106885	7.15E-07	2.88E-02
Hapmap50053-BTA-61516^b^	26	39018261	*C26H10orf84*	28724	1.11E-06	4.46E-02

Note: See note to Table 4.

**Table 7 pone-0013661-t007:** Genome-wise significant (p<0.05) SNPs with fat percentage (FP).

SNP	Chr.	Position (bp)	Nearest gene		Raw *P* value	Adjusted *P* value
			Name	Distance (bp)		
*Hapmap39717-BTA-112973* (rs41617243)	2	27529202	*KBTBD10*	Within	1.80E-07	7.24E-03
Hapmap51303-BTA-74377^b^ (rs41652649)	5	89694749	*ITPR2*	Within	1.20E-06	4.83E-02
BTB-00285653^b^ (rs43499009)	8	31663727	*NFIB*	Within	2.41E-07	9.69E-03
BFGL-NGS-119907^ab^	11	106766451	*GFI1B*	15556	1.30E-06	5.23E-02
ARS-BFGL-NGS-26919^a^	11	107216562	*LOC526069*	Within	6.87E-08	2.69E-03
Hapmap30381-BTC-005750^ab^	14	50872	*C14H8orf33*	23701	2.28E-18	9.17E-14
Hapmap30383-BTC-005848^ab^	14	76703	*C14H8orf33*	87	1.05E-28	4.22E-24
BTA-34956-no-rs^ab^ (rs41630614)	14	101473	*LOC785799*	3479	2.37E-18	9.53E-14
ARS-BFGL-NGS-57820^ab^	14	236532	*FOXH1*	3396	1.82E-48	7.32E-44
ARS-BFGL-NGS-34135^ab^	14	260341	*CYHR1*	Within	1.71E-30	6.88E-26
ARS-BFGL-NGS-94706^ab^ (rs17870736)	14	281533	*VPS28*	Within	5.78E-30	2.32E-25
*ARS-BFGL-NGS-4939* ^ab^	14	443937	*DGAT1*	160	5.23E-64	2.10E-59
Hapmap52798-ss46526455^ab^ (rs41256919)	14	565311	*MAF1*	Within	4.56E-12	1.83E-07
*ARS-BFGL-NGS-71749* ^ab^	14	596341	*OPLAH*	3237	1.20E-11	4.83E-07
*ARS-BFGL-NGS-107379* ^ab^	14	679600	*LOC786966*	460	1.77E-45	7.12E-41
*ARS-BFGL-NGS-18365* ^ab^	14	741867	*LOC524939*	15242	2.13E-08	8.57E-04
*Hapmap25384-BTC-001997* ^b^	14	835054	*MAPK15*	Within	6.22E-08	2.50E-03
*Hapmap24715-BTC-001973* ^b^	14	856889	*MAPK15*	Within	2.74E-08	1.10E-03
*BTA-35941-no-rs* ^ab^ (rs41627764)	14	894252	*ZNF623*	8779	2.72E-15	1.09E-10
ARS-BFGL-NGS-101653^b^	14	931162	*EEF1D*	Within	8.84E-11	3.56E-06
*ARS-BFGL-NGS-26520* ^ab^	14	996982	*ZC3H3*	Within	3.94E-14	1.58E-09
*UA-IFASA-6878* ^ab^ (rs41629750)	14	1044041	*GRINA*	15662	1.69E-13	6.80E-09
*ARS-BFGL-NGS-22866* ^b^	14	1131952	*LYPD2*	2653	3.30E-10	1.33E-05
*Hapmap25486-BTC-072553* ^ab^	14	1285037	*GML*	Within	1.09E-13	4.38E-09
*Hapmap29758-BTC-003619* ^ab^	14	1339276	*CYP11B1*	36652	1.95E-08	7.84E-04
*Hapmap30646-BTC-002054* ^ab^	14	1461085	*GPIHBP1*	1295	6.30E-20	2.53E-15
*Hapmap30086-BTC-002066* ^ab^	14	1490178	*ZNF66*	1566	6.61E-20	2.66E-15
*Hapmap30374-BTC-002159* ^ab^	14	1546591	*RHPN1*	Within	5.44E-13	2.19E-08
*ARS-BFGL-NGS-74378* ^b^	14	1889210	*GPR20*	71	1.46E-08	5.87E-04
*BFGL-NGS-117542* ^b^	14	1913108	*GPR20*	23969	7.64E-10	3.07E-05
*ARS-BFGL-NGS-33248* ^ab^	14	2130912	*PTK2*	Within	1.26E-08	5.07E-04
*UA-IFASA-9288* ^b^ (rs41624797)	14	2201870	*PTK2*	Within	2.19E-12	8.81E-08
*ARS-BFGL-NGS-22111* ^ab^	1	2347219	*EIF2C2*	25806	6.59E-08	2.65E-03
*UA-IFASA-7269* ^ab^ (rs41576704)	14	2370256	*EIF2C2*	2769	1.02E-08	4.10E-04
*Hapmap26072-BTC-065132* ^b^	14	2391826	*EIF2C2*	Within	2.15E-08	8.65E-04
*ARS-BFGL-NGS-56327* ^b^	14	2580414	*NIBP*	Within	1.58E-08	6.35E-04
*ARS-BFGL-NGS-100480* ^ab^	14	2607583	*NIBP*	Within	9.14E-16	3.68E-11
*UA-IFASA-5306* ^b^ (rs55617160)	14	2711615	*NIBP*	Within	2.12E-13	8.53E-09
*UA-IFASA-5765* ^a^	14	2763657	*NIBP*	Within	5.82E-07	2.28E-02
*ARS-BFGL-BAC-25166* ^a^	14	2805785	*NIBP*	Within	5.11E-08	2.00E-03
*Hapmap27703-BTC-053907* ^ab^	14	2826073	*NIBP*	Within	3.03E-11	1.22E-06
*Hapmap22692-BTC-068210* ^b^	14	3018726	*KCNK9*	25312	2.08E-07	8.37E-03
*Hapmap23302-BTC-052123* ^b^	14	3099635	*KCNK9*	106221	2.03E-07	8.16E-03
*Hapmap25217-BTC-067767* ^b^	14	3189312	*KCNK9*	195898	1.04E-07	4.18E-03
*UA-IFASA-6329* ^ab^ (rs41579243)	14	3465237	*COL22A1*	9864	4.29E-13	1.73E-08
*ARS-BFGL-NGS-3571* ^ab^	14	3587018	*COL22A1*	Within	5.38E-09	2.16E-04
*BFGL-NGS-118478* ^a^	14	3660264	*COL22A1*	Within	2.92E-07	1.14E-02
*BFGL-NGS-110563* ^ab^	14	3799228	*COL22A1*	84554	3.31E-16	1.33E-11
*Hapmap32262-BTC-066621* ^b^	14	3834069	*LOC618755*	67321	3.92E-11	1.58E-06
*BFGL-NGS-115947* ^b^	14	3865962	*LOC618755*	35428	3.79E-07	1.52E-02
*Hapmap30091-BTC-005211* ^b^	14	3940998	*LOC618755*	Within	2.10E-07	8.45E-03
*Hapmap27709-BTC-057052* ^ab^ (rs42305942)	14	4276966	*LOC100138440*	38554	4.14E-07	1.67E-02
*Hapmap51646-BTA-86764* ^b^ (rs41657812)	14	4302229	*LOC100138440*	13291	7.91E-08	3.18E-03
*Hapmap26591-BTC-056596* ^b^	14	4477036	*LOC100138440*	157701	6.62E-07	2.66E-02
*Hapmap23618-BTC-056528* ^b^ (rs42310935)	14	4518666	*LOC100138440*	199331	2.06E-08	8.29E-04
*Hapmap30988-BTC-056315* ^ab^	14	4693901	*LOC100138440*	374566	2.88E-08	1.16E-03
*UA-IFASA-6228* ^b^	14	5204594	*KHDRBS3*	560215	8.09E-07	3.25E-02
*ARS-BFGL-BAC-20965* ^b^	14	5225004	*KHDRBS3*	539805	2.77E-07	1.11E-02
*BFGL-NGS-110894* ^b^	14	5282438	*KHDRBS3*	482371	9.21E-09	3.70E-04
*Hapmap33635-BTC-049051* ^b^	14	5318261	*KHDRBS3*	446548	3.64E-07	1.46E-02
*Hapmap23851-BTC-048718* ^b^	14	5387836	*KHDRBS3*	376973	1.26E-06	5.07E-02
*Hapmap32234-BTC-048199* ^ab^	14	5640338	*KHDRBS3*	124471	3.82E-16	1.54E-11
*UA-IFASA-6647* ^ab^	14	5808644	*KHDRBS3*	Within	2.29E-14	9.21E-10
*Hapmap32948-BTC-047992* ^b^	14	5839290	*KHDRBS3*	Within	3.71E-07	1.49E-02
*ARS-BFGL-BAC-8730* ^ab^	14	6252101	*MIRN30D*	221420	7.03E-13	2.83E-08

Note: See note to Table 4.

**Table 8:** Genome-wise significant (p<0.05) SNPs with protein percentage (PP).

SNP	Chr.	Position (bp)	Nearest gene		Raw *P* Value	Adjusted *P* value
			Name	Distance (bp)		
Hapmap48524-BTA-92140^b^ (rs42552739)	5	80965296	*NCF4*	24749	3.69E-11	1.48E-06
ARS-BFGL-NGS-21133^b^	5	81003368	*CSF2RB*	2032	3.29E-07	1.32E-02
*Hapmap59369-rs29018333* ^b^ (rs29018333)	6	33989255	*LOC536367*	Within	1.52E-08	6.11E-04
*Hapmap24324-BTC-062449* ^b^	6	37024132	*HERC3*	6738	2.33E-17	9.37E-13
*BTA-121739-no-rs* ^b^ (rs41622323)	6	37454409	*PKD2*	Within	7.08E-07	2.85E-02
*BTB-00251047* ^b^ (rs43463988)	6	41928694	*LOC100140991*	401634	4.33E-07	1.74E-02
*Hapmap41083-BTA-76098* ^b^ (rs41652041)	6	80715299	*LOC100140587*	4319	1.17E-06	4.71E-02
*ARS-BFGL-NGS-57820* ^ab^	14	236532	*FOXH1*	3396	2.82E-08	1.13E-03
ARS-BFGL-NGS-34135^ab^	14	260341	*CYHR1*	Within	3.88E-09	1.56E-04
ARS-BFGL-NGS-94706^ab^ (rs17870736)	14	281533	*VPS28*	Within	2.28E-08	9.17E-04
*ARS-BFGL-NGS-4939* ^ab^	14	443937	*DGAT1*	160	3.73E-08	1.50E-03
*ARS-BFGL-NGS-107379* ^ab^	14	679600	*LOC786966*	460	7.73E-07	3.11E-02
*UA-IFASA-6878* ^b^ (rs41629750)	14	1044041	*GRINA*	15662	6.79E-07	2.73E-02
*Hapmap27703-BTC-053907* ^a^	14	2826073	*NIBP*	Within	3.95E-07	1.55E-02
*BFGL-NGS-118998* ^ab^	20	34036832	*GHR*	Within	7.87E-07	3.17E-02
*ARS-BFGL-BAC-2469* ^b^ (rs41937533)	20	35552477	*LOC518808*	22514	1.21E-08	4.87E-04
*BTA-50402-no-rs* ^b^ (rs41945918)	20	36668000	*LOC782462*	19510	7.57E-07	3.04E-02
*Hapmap57531-rs29013890* ^b^ (rs29013890)	20	36955575	*LOC782833*	32019	8.97E-08	3.61E-03
*BTB-00778154* ^ab^ (rs41941646)	20	37399087	*C9*	10219	6.21E-07	2.50E-02
*BTB-00778141* ^ab^ (rs41941633)	20	37442583	*FYB*	35360	1.91E-11	7.68E-07
*ARS-BFGL-NGS-38482* ^b^	20	37708167	*RICTOR*	Within	1.99E-08	8.00E-04
*Hapmap39660-BTA-50453* ^b^ (rs41581059)	20	37865657	*LOC100138964*	Within	2.97E-07	1.19E-02
ARS-BFGL-NGS-22355^b^	20	38899763	*GDNF*	17831	5.65E-07	2.27E-02
BTB-00782435^b^ (rs41942492)	20	39485917	*NIPBL*	Within	1.02E-07	4.10E-03
BTA-13793-rs29018751^b^ (rs29018751)	20	39518857	*NIPBL*	Within	6.27E-10	2.52E-05
BTB-01842107^b^ (rs42954630)	20	39601103	*NIPBL*	Within	1.53E-10	6.15E-06
Hapmap53199-rs29014437^a^ (rs29014437)	20	39728147	*LOC782284*	38124	3.35E-07	1.35E-02
BTA-102910-no-rs^b^ (rs41574319)	20	41947097	*RAI14*	171370	2.64E-07	1.06E-02

Note: See note to Table 4.

#### Milk Yield (MY)

As seen from Table 4, 14 out of 20 SNPs are located within a 3.63 Mb segment (between 0.07 and 3.7 Mb) on BTA 14. Ten of them fall into the regions that have been reported to harbor QTL for MY [5], [21], [28], [29], [30], [31], [32]. Furthermore, 6 of these SNPs are harbored within the regions of known genes, and the others are located 87 to 84,554 bp away from the nearest known genes.

#### Fat Yield (FY)

As presented in Table 5, 6 out of 9 SNPs are clustered within a 0.55 Mb segment (between 0.05 and 0.6 Mb) on BTA 14. Eight out of them fall in the regions which have been reported to harbor QTL for FY previously [5], [28], [31], [33], [34], [35]. Furthermore, two of these SNPs fall within the regions of known genes, and the others are located 160 to 285,289 bp away from the nearest known genes.

#### Protein Yield (PY)

As shown in Table 6, among these 21 SNPs, 7 out of them are located within a 3.33 Mb segment (between 0.07 to 3.4 Mb) on BTA 14. Further, 14 out of these SNPs are within the QTL regions for PY reported in previous studies [5], [21], [28], [33], [36], [37], [38], [39]; 5 of them are located within the regions of known genes, and the others are located 87 to 385,764 bp away from the nearest known genes.

#### Fat Percentage (FP)

From Table 7, 60 are located within a 6.2 Mb segment (between 0.05 to 6.25 Mb) on BTA 14. 53 of them are located within the QTL regions for FP reported in previous studies [5], [34], [39], [40], [41], [42], [43], [44]. Further, 27 of the 65 detected SNPs are located within the regions of known genes, and the others are 71 to 560,215 bp away from the nearest known genes.

#### Protein Percentage (PP)

As given by Table 8, out of 28 identified SNPs, there are 4, 7, and 14 SNPs located within a 8.0 Mb segment (between 33.9 to 41.9 Mb) on BTA6, a 2.59 Mb segment (between 0.23 to 2.82 Mb) on BTA14, and a 7.9 Mb segment (between 34.0 to 41.9 Mb) on BTA20, respectively. Among these 28 SNPs, 17 are located within the QTL regions for PP identified in previous studies [5], [28], [29], [34], [42], [45]. Further, 11 of these 28 SNPs are located within the regions of known genes, and the others are 160 to 401,634 bp away from the nearest known genes.

### Population stratification assessment

**Figure 2:**
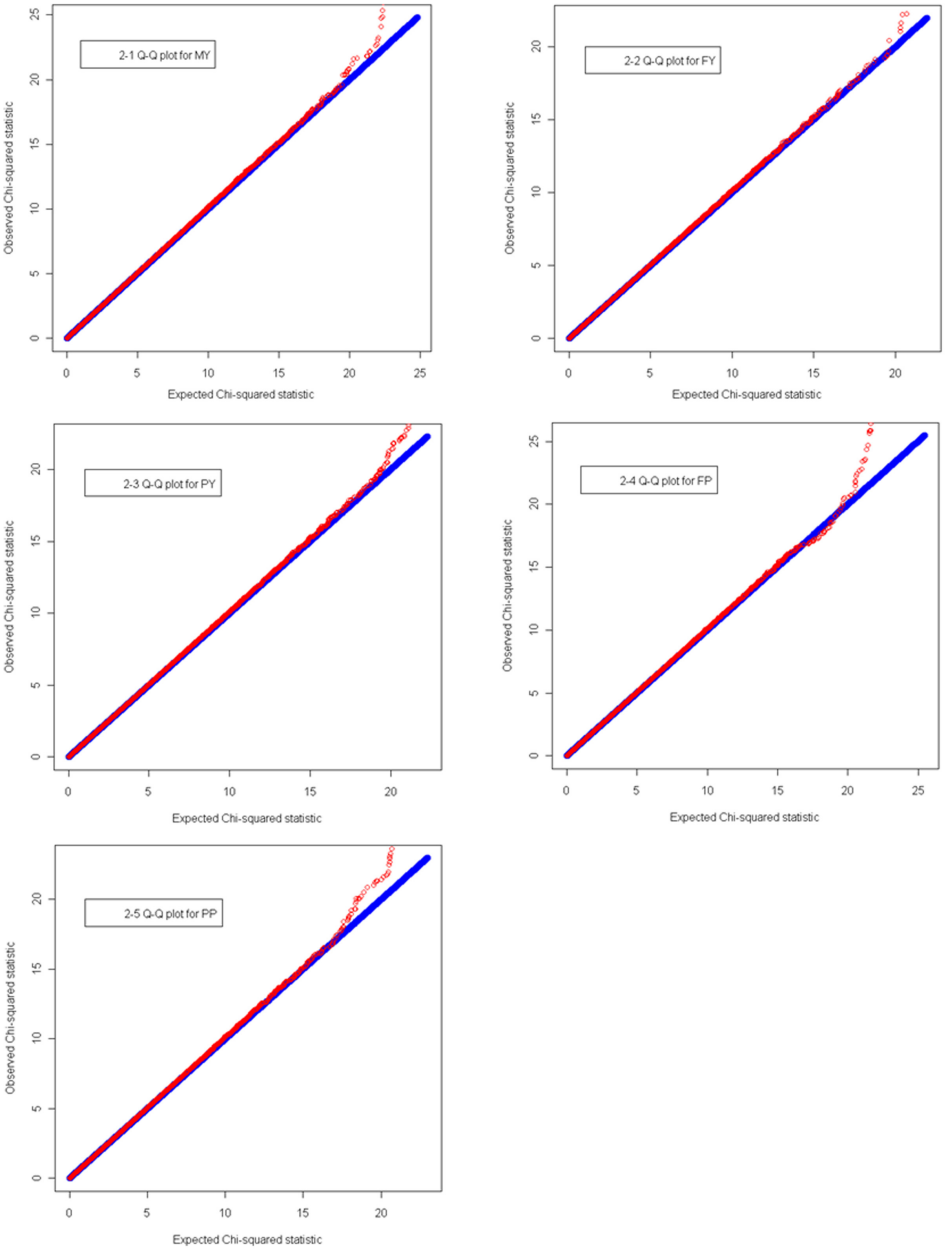
Quantile-quantile (Q-Q) plots of genome-wide association results by MMRA for five milk production traits. Under the null hypothesis of no association at any SNP locus, the points would be expected to follow the slope lines. Deviations from the slope lines correspond to loci that deviate from the null hypotheses.

The “Q-Q” plots for the test statistics of MMRA are shown in Fig. 2-1 to 2-5. From these plots, it is apparent that the distributions of the *χ^2^* statistics generated from the association tests across the SNPs tested show no evidence of overall systematic bias. That is, the observed *χ^2^* statistics of the significant SNPs are above the expected *χ^2^* statistics, which are largely at the adjusted genome-wide significance level. The profiles of the Q-Q plots clearly show that the significant SNPs identified by MMRA are unlikely threaten by potential population stratification.

## Discussion

In this study, we performed a GWA study for five milk production traits using a daughter design in Chinese Holstein population. To our knowledge, this is one of the first GWA studies for milk production traits using the Illumina BovineSNP50 BeadChip. Two statistical methods, L1-TDT and MMRA, were implemented to analyze association between SNPs and phenotypes. These two methods belong to two distinct analytical approaches, *i.e.*, family-based (L1-TDT) and population-based (MMRA) approaches, respectively, both of which have been widely employed in GWAS. Comparisons between the two methods have been well conducted by many investigators [27], [46], [47], [48]. Consensus with respect to their performance is twofold. On the one hand, population-based analyses largely outperform family-based analyses in statistical power and efficiency. The power limitation of family-based analyses results from “overmatching” on genotype [49]. Much fewer significant SNPs detected by L1-TDT compared with MMRA in this study present consistent evidence for this aspect in practice. On the other hand, family-based analysis always guards against population admixture/stratification caused by recent migration and/or non-random mating, and do not give spurious significant results, although at the expense of some loss of power [50]. The “Q-Q” plots for the test statistics of MMRA (Fig. 2-1 to 2-5) demonstrate that no population admixture/stratification exists in our population. Therefore, it is safe to declare that the SNPs detected by MMRA as well as L1-TDT have convincing associations with the traits of interest.

BTA14 has been received wide attention by many investigators. Apart from a large number of QTL reported on BAT14 [34], [39], [51], [52], the well-known *DGAT1* gene[5] located at ∼0.44Mb is generally accepted as a major gene affecting milk production traits. Bennewitz *et al.* [28] revisited the QTL on BTA14 and concluded that there should exist a further conditional QTL which should be in linkage with the *DGAT1* gene, and possible epistatic effects arising from them may be an additional source of genetic variation for milk production traits. Indeed, Kaupe *et al.* [53] recently reported that the *CYP11B1* gene located at ∼1.33Mbp has significant effects on MY, PY, FP and PP, and the allele substitution effects of *CYP11B1* and *DGAT1* together explained more variation in milk production traits than *DGAT1* alone. In our study, an apparent feature of our findings is that a large proportion of the significant SNPs (61 out of 105) are located on BTA14. Of the 61 SNPs, 59 are located within the reported QTL regions. In particular, all segments on BTA14 which harbor multiple SNPs for the five traits also harbor the *DGAT1* gene, and the four segments for MY, PY, FP and PP also harbor the *CYP11B1* gene. Within these segments, 13 SNPs are located very close (within 1Mb) to the *DGAT1* gene with the closest one (ARS-BFGL-NGS-4939) only 160bp away from it and 14 SNPs very close to the *CYP11B1* gene with the closest one (Hapmap25486-BTC-072553 ) only 8,693bp away from it.

In addition to the SNPs on BTA14, most (27 out of 44) of the significant SNPs on other chromosomes are also located within the reported QTL regions. Further, some SNPs are also within or close (within 1Mb) to the reported candidate genes (for a summary of cattle candidate genes for milk production traits, see [54]). In particular, a SNP (BFGL-NGS-118998) located at 34,036,832 bp on BTA20 was found to fall within the *GHR* gene, which is also generally accepted as a functional causal gene affecting milk yield and components [5], [6]. The other SNPs include the SNPs BTA-121739-no-rs and Hapmap24324-BTC-062449 on BTA6, which are 20,591bp and 450,868bp away from the *ABCG2* gene [55], respectively, and the SNP ARS-BFGL-NGS-26919 on BTA11, which is 41,562bp away from the *LGB* gene [56].

It is notable that for either L1-TDT or MMRA some detected SNPs are associated with phenotypic variation in multiple production traits, including the SNPs ARS-BFGL-NGS-4939, ARS-BFGL-NGS-57820, and ARS-BFGL-NGS-107379 on BTA14 (for all of the five traits), the SNPs ARS-BFGL-NGS-94706 and ARS-BFGL-NGS-34135 on BTA14 (for MY, FY, FP, and PP), the SNPs Hapmap30383-BTC-005848, Hapmap30646-BTC-002054, ARS-BFGL-NGS-100480, and UA-IFASA-6329 on BTA14 (for MY, PY, and FP), the SNP UA-IFASA-6878 on BTA14 (for MY, FP, and PP), the SNPs Hapmap52798-ss46526455, BFGL-NGS-110563, Hapmap25486-BTC-072553, and Hapmap30086-BTC-002066 on BTA14 (for MY and FP), the SNPs ARS-BFGL-NGS-91705 on BTA1, Hapmap38643-BTA-95454 on BTA3, BFGL-NGS-110018 on BTA5, and Hapmap50053-BTA-61516 on BTA26 (for MY and PY), the SNPs Hapmap30381-BTC-005750 on BTA14 (for FY and FP), and the SNP Hapmap27703-BTC-053907 on BTA14 (for FP and PP). This could be explained by pleiotropic effects of these SNPs on multiple milk production traits, leading to genetic correlations among them and there were similar result in many prior studies [28], [53].

In this study, we performed GWAS in the way of SNP by SNP individually via regressing the observations of a single trait on either the genotypes of a SNP (MMRA) or the allele transmission patterns of a SNP from bulls to corresponding half-sib offspring (L1-TDT). Previous studies have shown that single marker tests provide similar or greater power than haplotype-based approaches [57], [58]. In contrast to haplotype-based methods, the main advantage of the single locus test is that it does not necessitate information of SNP positions and reconstruction haplotypes of multiple SNP loci. Thus, it is the preferable method for large scale genome-wise association analyses, *e.g.*, GWAS. Also, we individually perform GWAS for each of five milk production traits. This is the most conventional strategy for current GWAS. However, the five milk production traits considered here are generally regarded as correlated and thus should share common environmental/genetic factors. A multiple traits instead of single trait analysis may be a promising way to take correlations among these traits into consideration. Multivariate analyses have been widely adopted in linkage studies [59], [60], [61], [62], [63], and it has been generally accepted that multivariate analyses outperform univariate analyses in terms of increasing statistical power and precision of parameter estimation [64], [65]. In the next step, an optimal multiple traits analytical strategy will be pursued to further enhance our GWA studies.

In our study, the EBVs of daughters were used as phenotypes for association analysis. Besides EBVs, yield deviation (YD) and de-regressed EBVs of individuals are also commonly used as phenotypic observations in GWAS as well as in LA and LA/LD analyses for milk production traits. Comparison among these three kinds of phenotypes with respect to their influence on QTL mapping [66] and marker assisted selection studies [67] demonstrate that none of them has absolute advantages over the others. We also compared using EBVs and de-regressed EBVs as phenotypes for our GWAS and it turned out that the findings of them are basically overlap (data not shown). Therefore, only the findings from using EBVs are reported herein.

In all, the present study revealed 105 genome-wise significant SNPs for milk production traits in Chinese dairy cattle population using two different association analysis approaches (L1-TDT and MMRA). Most of these SNPs (86 out of 105) are located within the previously reported QTL regions, and some within or close to the reported candidate genes. The general consistence of the significant SNPs detected herein with the reported QTL and candidate genes and the agreement of the results of the two analysis approaches present strong support for the outcomes of this study. Our findings herein lay a preliminary foundation for guiding follow-up replication studies, and eventually revealing the causal mutations underlying milk production traits in dairy cattle.
